# Novel Families of Archaeo-Eukaryotic Primases Associated with Mobile Genetic Elements of Bacteria and Archaea

**DOI:** 10.1016/j.jmb.2017.11.014

**Published:** 2018-03-02

**Authors:** Darius Kazlauskas, Guennadi Sezonov, Nicole Charpin, Česlovas Venclovas, Patrick Forterre, Mart Krupovic

**Affiliations:** 1 Institute of Biotechnology, Vilnius University, Saulėtekio av. 7, Vilnius 10257, Lithuania; 2 Sorbonne Universités, UPMC Université Paris 06, CNRS, UMR 7138 Evolution Paris Seine—Institut de Biologie Paris Seine, Paris 75005, France; 3 Unité Biologie Moléculaire du Gène chez les Extrêmophiles, Department of Microbiology, Institut Pasteur, 25 rue du Docteur Roux, Paris 75015, France

**Keywords:** *Thermococcus* plasmids, DNA replication, evolution, helicases, structural modeling, AEPs, archaeo-eukaryotic primases, LigD, ligase D, NHEJ, non-homologous end joining, HTH, helix-turn-helix, PriCT-1/PriCT-2, Primase-C Terminal-1/2, MGEs, mobile genetic elements, WH, winged-helix, ZnBD, Zn-binding domain, DUF, domain of unknown function, pVOG, prokaryotic virus orthologous groups

## Abstract

Cellular organisms in different domains of life employ structurally unrelated, non-homologous DNA primases for synthesis of a primer for DNA replication. Archaea and eukaryotes encode enzymes of the archaeo-eukaryotic primase (AEP) superfamily, whereas bacteria uniformly use primases of the DnaG family. However, AEP genes are widespread in bacterial genomes raising questions regarding their provenance and function. Here, using an archaeal primase–polymerase PolpTN2 encoded by pTN2 plasmid as a seed for sequence similarity searches, we recovered over 800 AEP homologs from bacteria belonging to 12 highly diverse phyla. These sequences formed a supergroup, PrimPol-PV1, and could be classified into five novel AEP families which are characterized by a conserved motif containing an arginine residue likely to be involved in nucleotide binding. Functional assays confirm the essentiality of this motif for catalytic activity of the PolpTN2 primase–polymerase. Further analyses showed that bacterial AEPs display a range of domain organizations and uncovered several candidates for novel families of helicases. Furthermore, sequence and structure comparisons suggest that PriCT-1 and PriCT-2 domains frequently fused to the AEP domains are related to each other as well as to the non-catalytic, large subunit of archaeal and eukaryotic primases, and to the recently discovered PriX subunit of archaeal primases. Finally, genomic neighborhood analysis indicates that the identified AEPs encoded in bacterial genomes are nearly exclusively associated with highly diverse integrated mobile genetic elements, including integrative conjugative plasmids and prophages.

## Highlights

• Primases of the archaeo-eukaryotic primase (AEP) superfamily are widespread in bacteria.
• We describe five new AEP families in bacteria belonging to 12 diverse phyla.
• The new AEP families display a conserved signature motif likely involved in nucleotide binding.
• The primase domains are fused to diverse functional domains, revealing new families of putative helicases.
• The novel primases are encoded within highly diverse integrated mobile genetic elements.

## Graphical Abstract

**Figure:**
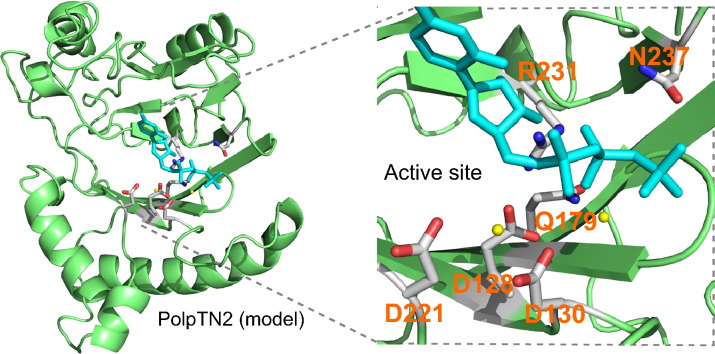

## Introduction

In all cellular organisms, replicative DNA polymerases cannot initiate DNA synthesis *de novo*. Thus, a second enzyme, called DNA primase, is necessary for generating an RNA primer, which is subsequently extended by the DNA polymerase. Bacteria encode primases of the DnaG family, which have the TOPRIM fold also found in type IA and II topoisomerases, OLD family nucleases, and RecR proteins [1]. By contrast, archaea and eukaryotes encode homologous heterodimeric primases evolutionarily unrelated to DnaG and consisting of the small catalytic (PriS) and the large regulatory (PriL) subunits. The former contains a highly derived version of the RNA recognition motif fold, which is also present in viral RNA-dependent RNA polymerases, reverse transcriptases, cyclases, and DNA polymerases of the A/B/Y families [2], [3], whereas the PriL consists of two largely α-helical domains, where the C-terminal domain contains a 4Fe–4S cluster [4], [5], [6].

Although archaeo-eukaryotic primases (AEPs) are not directly involved in bacterial DNA replication, many bacteria encode AEP homologs, some of which have important well-defined cellular functions. For example, one group of bacterial AEP constitutes an integral component of the bacterial ligase D (LigD) complex responsible for the non-homologous end joining (NHEJ) pathway of double-strand break repair [7], [8]. Furthermore, AEPs are not restricted to cellular organisms but are also encoded by viruses infecting hosts in all three domains of life as well as some plasmids. Unexpectedly, a global survey of DNA replication proteins from double-stranded (ds) DNA viruses has revealed that bacteriophages encode AEP nearly as frequently as the DnaG-like enzymes, raising questions regarding their provenance [9]. In viruses and plasmids, the AEP domain is often fused to other functional domains, most notably various helicases, Zn-binding and helix-turn-helix (HTH) DNA-binding domains as well as α-helical domains known as Primase-C Terminal-1 (PriCT-1) and PriCT-2 [3], [9]. Although AEP proteins are capable of primer extension *in vitro* (i.e., DNA polymerase activity), the lack of proof-reading capacity of all characterized members of this superfamily suggests that they predominantly act as primases *in vivo*.

Based on sequence comparisons and the presence of characteristic motifs, AEP have been classified into 13 different families, 12 of which can be further grouped into 3 major clades: (i) the AEP proper clade, including the archaeal and eukaryotic PriS proteins, NHEJ primases, and Lef-1-like primases of baculoviruses; (ii) NCLDV–herpesvirus primase clade, including primases of nucleocytoplasmic large DNA viruses (NCLDV) and herpesviruses; and (iii) Prim-Pol clade, including Prim-Pol proper family, *Escherichia coli* Z1568-like family, *Deinococcus* DR0530-like family, *Anabaena* all3500-like family, *Bradyrhizobium* bll5242-like family, ColE2 Rep-like family, and RepE/RepS family, and finally, BT4734-like family, which is not associated with any clade [3], [7]. All these families share a set of three conserved motifs (I, II, and III). Motifs I (hhhDhD/E, where “h” is a hydrophobic residue) and III (hD/E) are involved in divalent metal ion coordination for catalysis, whereas motif II (sxH, where “s” is a small residue and “x” is any residue) is required for nucleotide binding [3], [7]. Multiple mutagenesis studies have shown that these motifs are essential for catalysis [10], [11], [12], [13], [14], [15].

Recently, several new AEP enzymes have been reported, including TthPrimPol from *Thermus thermophilus* [16], human PrimPol [17], and PrimPol from a deep-sea vent phage NrS-1 [18]; however, their position within the global AEP superfamily remains unclear. In addition, Burroughs and Aravind [19] have recently described several families of divergent AEP domains found as components of multidomain proteins predicted to function as RNA-repair systems, primarily in kinetoplastids. Finally, we have identified and biochemically characterized a divergent AEP, PolpTN2, encoded by pTN2-like plasmids of hyperthermophilic archaea of the order Thermococcales [20], [21], [22]. Interestingly, in PolpTN2, the PriS-like AEP domain is fused to a large C-terminal domain, which contains the predicted 4Fe–4S cluster and is similar to the archaeal and eukaryotic PriL subunits [20]. Similarly to other AEP proteins [7], PolpTN2 exhibits primase, polymerase, and nucleotidyl transferase activities and specifically incorporates dNTPs to the exclusion of rNTPs. Uniquely, the N-terminal domain possesses reverse transcriptase activity. Furthermore, sequence analysis has shown that whereas the three acidic active site residues (motifs I and III) are conserved in PolpTN2, the signature active site histidine (motif II) is replaced by glutamine [20], suggesting that PolpTN2 might be a prototype of a new AEP family. Here, we explore the position of PolpTN2 and other recently described PrimPol proteins within the global AEP superfamily and describe a new supergroup, PrimPol-PV1, consisting of several AEP families widespread in diverse bacterial and archaeal plasmids and viruses.

## Results and Discussion

### Novel AEP families

**Fig. 1:**
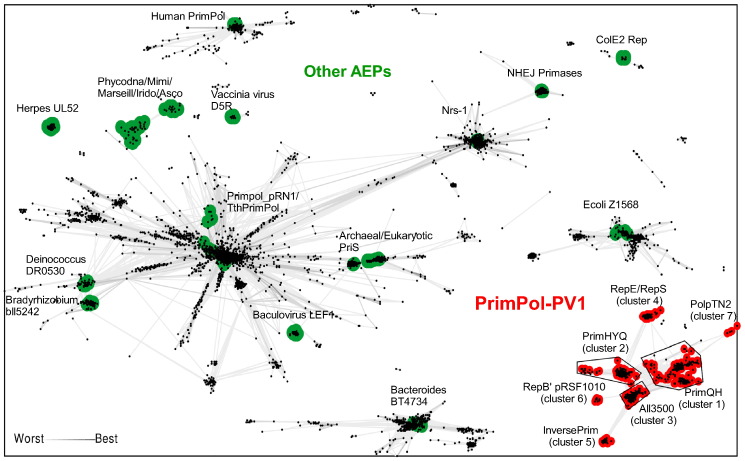
Global diversity of AEP proteins. Protein sequences were clustered by the pairwise sequence similarity (CLANS *P*-value ≤ 1e − 11). Representative members of well-characterized AEP groups are shown in green; PrimPol-PV1, red. Zoom-in on the PrimPol-PV1 group is shown in Fig. S1.

To identify homologs of the PolpTN2 primase domain and to explore their relationship to members of the previously established AEP families [3], we performed iterative searches (seven iterations) seeded with the AEP domain of PolpTN2 as well as with the representative members of each of the established AEP families using Jackhmmer [23]. In addition, the data set was supplemented with several AEP groups, representing the recently published TthPrimPol [16], human PrimPol [17], and PrimPol from the NrS-1 phage [18]. The data set was clustered based on all-against-all sequence similarity using CLANS [24] (see Materials and Methods), with the *P*-value cutoff (1e − 11) being empirically selected to optimize the separation of the previously defined AEP families. It should be noted that due to high divergence and short length (~ 200 aa) of the AEP domain, a meaningful phylogenetic analysis of the AEP superfamily is not feasible. The clustering and subsequent community (or cluster) detection analysis confirmed the distinctiveness of the previously defined AEP families (Fig. 1). Furthermore, whereas the TthPrimPol-like and NrS-1-like primases grouped with pRN1-like primases from the PrimPol proper family, human PrimPol-like AEP did not merge into any of the existing families and should be considered as an independent new family within the AEP superfamily (Fig. 1). As reported previously [20], close homologs of PolpTN2 are restricted to members of the archaeal order Thermococcales; consistently, in our analysis, these sequences form a separate cluster. Nevertheless, the PolpTN2-like cluster is linked to three highly diverse, interconnected groups (clusters 1–3) and three smaller, more distantly related clusters connected to them (referred to as InversePrim, RepB′, and RepE/RepS in Figs. 1 and S1). Notably, two of these groups were previously denoted as distinct AEP families, namely, the RepE/RepS family and the *Anabaena* all3500-like family [3], the latter corresponding to cluster 3. Throughout this article, we retain the original names of these two families. By contrast, the five other clusters, including the PolpTN2-like group, in previous studies were not formally classified and are henceforth considered as novel AEP families. Collectively, the PolpTN2 family and the six clusters of interrelated homologs form a supergroup of diverse AEP, which we collectively refer to as PrimPol-PV1 supergroup (see below). In the next sections, we present comparative characterization of the seven families clustering with the PolpTN2, focusing on their sequence conservation patterns, taxonomic distribution, and domain organizations. We also note that the new AEP families recently described by Burroughs and Aravind [19] in eukaryotes are not appreciably similar to members of the PrimPol-PV1 supergroup found in bacteria and archaea, and are thus not further considered in the present work.

### New AEP families show distinct conservation patterns in motif II

**Fig. 2:**
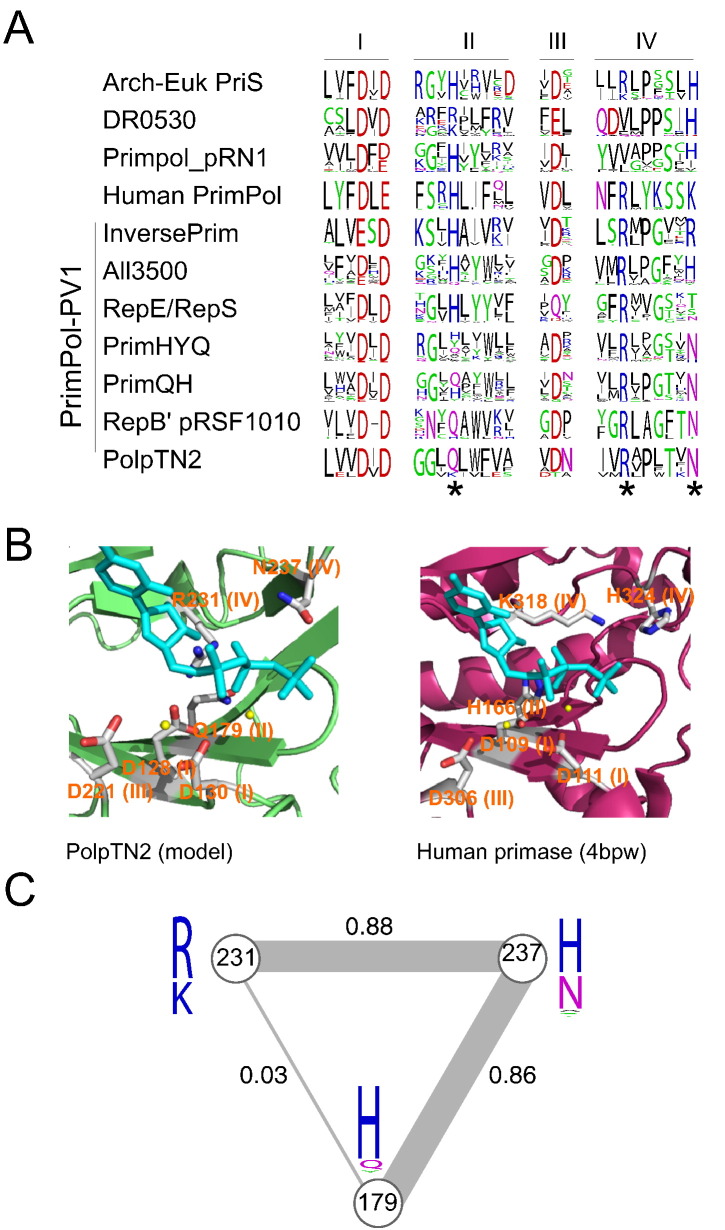
Comparison of conserved motifs across different AEP families. (A) Alignment of sequence logos from PolpTN2 homolog families and PriS from archaea and eukaryotes. Residues are colored by their chemical properties (polar, green; basic, blue; acidic, red; hydrophobic, black; neutral, purple). Residues thought to be involved in nucleotide binding in PolpTN2 are starred. (B) Active sites of PolpTN2 and human primase. Model of PolpTN2 is aligned with the structure of human primase (PDB: 4bpw). Metal ions are shown as yellow spheres. Incoming UTP is shown in cyan. (C) Covariation of residues in the PrimPol domain of PrimPol-PV1 and PriS homologs. The data set (1427 sequences) was collected by running 10 Jackhmmer iterations against nr70 database. Note that His residue at position 179 is primarily contributed by PriS homologs. PSICOV covariation score is given near the edges of triangle. Thickness of the line is proportional to the covariation score. WebLogos for each position are shown at the tips.

As mentioned above, members of the AEP superfamily contain 3 conserved catalytic motifs [3]. Analysis of the sequence conservation patterns in the PolpTN2-like group and the six related clusters (Fig. 1) showed that acidic residues in motifs I and III involved in divalent metal ion coordination are nearly universally conserved (Fig. 2A). The only exception is observed in the RepE/RepS family, where the conserved Asp in motif III is replaced by a Gln (Fig. 2A). We note that the Gln in most proteins of this family is preceded, albeit with a variable spacing, by Asp or Glu residues that might participate in the catalysis.

Unexpectedly, the conservation of the diagnostic His residue in motif II, which was previously shown to be essential for catalytic activities of many AEP enzymes [25], [26], [27], was found to be family specific. Members of the families *Anabaena* all3500-like, RepE/RepS, and InversePrim contain a characteristic His residue in motif II, whereas in PolpTN2-like and RepB′-like enzymes, the His is substituted with a conserved Gln (Fig. 2A). Interestingly, in sequences belonging to cluster 1, the same position is occupied by either Gln or His, whereas in cluster 2, there is even greater variation, with the His, Gln and Tyr residues being the most prevalent ones. The only other AEP family in which His is not present in motif II is the DR0530-like family. However, in the latter family, the His is replaced by other positively charged residues, namely, Arg or Lys (Fig. 2A). Based on the conservation patterns in motif II, we refer to clusters 1 and 2 as AEP families PrimQH and PrimHYQ, respectively (Fig. 1, Fig. 2A). The lack of conservation in motif II highlights the degree of variability within the AEP superfamily, where even the diagnostic motifs are amenable to change, and suggests that PolpTN2-like enzymes have evolved alternative solutions for nucleotide binding within the active site.

### Conserved Arg residue in a new motif IV is essential for PolpTN2 enzymatic activities

Analysis of the multiple sequence alignments revealed a previously unnoticed conserved *Rh* motif (where h stands for a hydrophobic residue) located downstream of motif 3 (henceforth referred to as motif IV; Fig. 2A), in the so-called flange region, a β-strand perpendicular to the plane of the RNA recognition motif sheet [3]. Although the corresponding Arg is not conserved throughout the AEP superfamily, it is invariantly present in the PolpTN2-like family and the six related families as well as in some other AEP families, most notably archaeal/eukaryotic PriS family (containing either Arg or Lys) and the human PrimPol family (Fig. 2A). In the human PriS, the corresponding residue (Lys318) has been implicated in nucleotide binding and its mutation to Ala abolished the RNA primer synthesis [25]. Structural studies have revealed that Lys318 extends its side chain underneath the ribose and phosphate groups, thereby assisting in NTP binding by charge neutralization [25].

To investigate the potential role of the Arg residue conserved in the PolpTN2-like family (and related families), we built a homology model of PolpTN2 (Fig. 2B) based on the X-ray structure of RepB′ encoded by plasmid RSF1010 [13] and modeled-in a nucleotide substrate using the nucleotide-bound human PriS structure as a template [25]. Analysis of the resulting structural model showed that the side chain of Arg in motif IV points toward the nucleotide bound in the active site (Fig. 2B), consistent with the assumption that this residue plays an important role in proper binding and orientation of the nucleotides. Notably, the role of the conserved Arg (or Lys in the case of primases) might be redundant with that postulated for the His residue in motif II. Thus, the absence of His residue in the motif II of PolpTN2-like, PrimQH and PrimHYQ family enzymes might be compensated by the conserved Arg in motif IV. To test this hypothesis, we analyzed covariation between the corresponding residues from 1427 sequences of PolpTN2 primase domain homologs using PSICOV (see Materials and Methods). It turned out that covariation between His (motif II) and Arg (motif IV) residue positions (PolpTN2, 179, and 231, respectively) is very low (covariation score of 0.03; Fig. 2C). However, each of the two positions showed a strong link to another nucleotide-binding residue within motif IV (PolpTN2, Asn237). Covariation scores of pairs 179 | 237 and 231 | 237 were among the top four results reported by PSICOV (Supplementary data file 2; Fig. 2). Furthermore, to substantiate these results, analysis was repeated using PLMC script from the EVmutation package. Results were similar: 231 | 237 and 179 | 237 pairs were among the top scorers (coupling scores 0.331 and 0.182, respectively; Supplementary data file 2) and coupling between positions 231 and 179 was much lower (0.047). The coevolution is most noticeable in the 179 | 237 pair, where histidine is most often paired with a positively charged residue and glutamine tends to go along with an asparagine (Fig. 2A). Most popular three-residue combination (179 | 231 | 237) was “HKH” (Figs. S2 and 2). These residues are found in eukaryotic PriS and primases from prokaryotes (Fig. S2, ProkHKH). Interestingly, some members of the latter group colocalize with bacterial Argonaute family proteins and subtype III-B CRISPR-Cas systems, but their functions remain unknown [28]. Surprisingly, although sequences of eukaryotic and archaeal PriS subunits are closely related, there was no archaeal PriS with “HKH” residue combination, which instead display the “HRH” signature (Fig. S2). Mutation of these positions in human PriS (H166, K318, and H324, respectively) abolishes or negatively affects RNA primer synthesis, and it has been suggested that these residues participate in the NTP binding [25]. Our observation that two of these three residue pairs coevolve together further supports the idea that they are functionally connected.

**Fig. 3:**
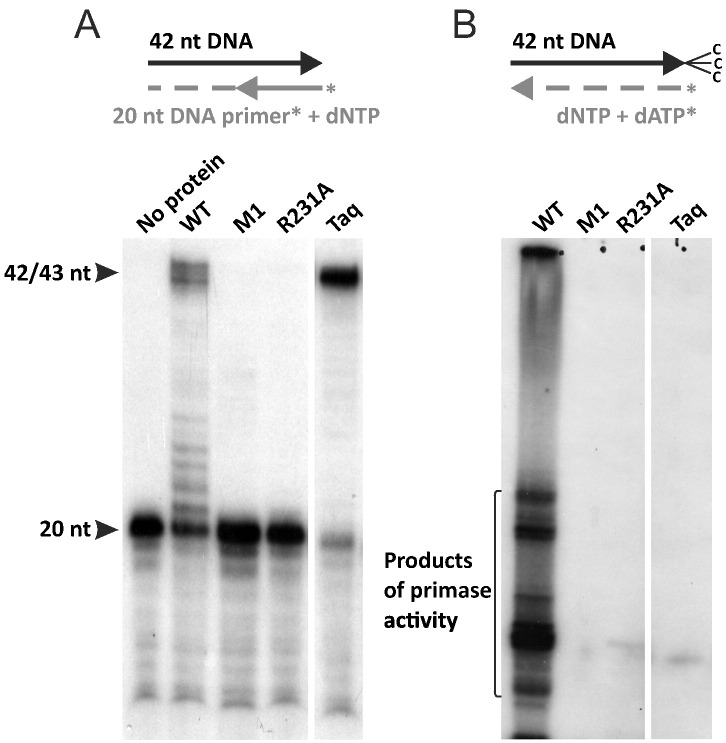
Catalytic activities of the wt and mutant PolpTN2 proteins. (A) The M1 and R231A mutants of PolpTN2 lost the DNA polymerase activity. The wild-type (lane 2) and two mutant forms of the recombinant protein PolpTN2 (lanes 3 and 4) or Taq polymerase (lane 5) were incubated with a short primer-template substrate (5^′^-^32^P-labeled 20-nt oligonucleotide hybridized to 42-nt template). In the control reaction with no protein added (lane 1), no primer elongation was observed. (B) The M1 and R231A mutants of PolpTN2 lost the dNTP-dependent primase/polymerase activity. Primase reactions were performed using 42-nt-long oligonucleotide template blocked at 3′ end with the 3′-Spacer C3 (indicated with 3 Cs). Lane 1, wt PolpTN2; lane 2, mutant M1; lane 3, mutant R231A; lane 4, Taq DNA polymerase; lanes 5 and 6, labeled length markers. The variation in the length of synthesized products in lane 1 could be due to the terminal transferase activity of the PolpTN2 [20].

To verify the importance of motif IV in the catalysis, we constructed a PolpTN2 mutant in which the Arg231 was substituted for an Ala (R231A). As a negative control, we used a mutant M1 in which the two catalytic Asp residues of the motif I were changed to Leu residues (DID → LIL). The two mutants, the wild-type (wt) PolpTN2 and Taq DNA polymerase, were assayed for the DNA polymerase and primase activities. Whereas the wt PolpTN2 was able to perform both reactions, the M1 mutant protein devoid of the catalytic Asp residues in motif I was inactive as expected. Interestingly, the R231A protein was equally incapable of synthesis of the expected products in both DNA polymerase (Fig. 3A) and primase (Fig. 3B) assays, whereas Taq DNA polymerase performed only DNA polymerization but not the priming reaction, as expected. This result in combination with the sequence conservation analysis indicates that, at least in PolPTN2, motif IV is essential for catalysis.

### Taxonomic distribution of the new AEP families

**Fig. 4:**
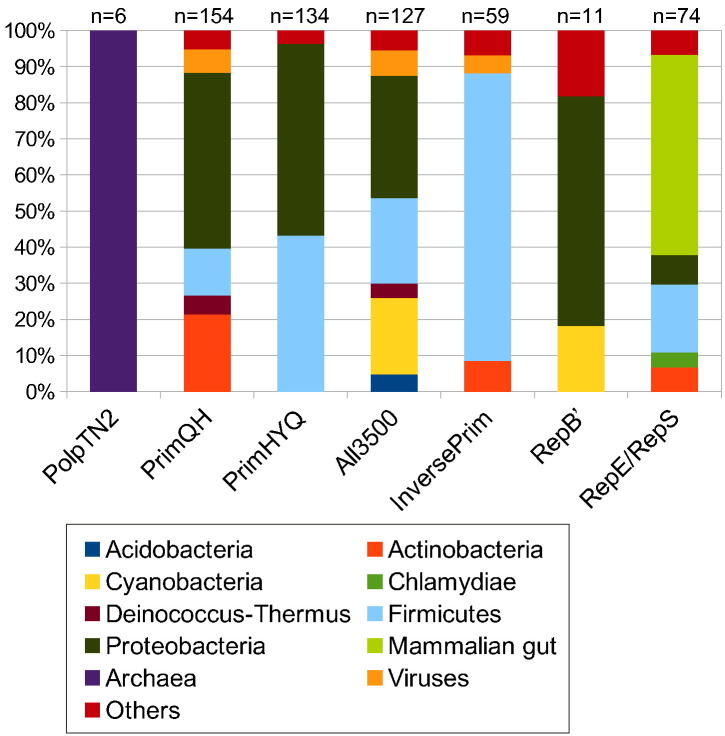
Taxonomic distribution of bacterial AEP of the PrimPol-PV1 supergroup. Taxa containing less than 4% (10% for RepB′) of representatives were merged into group “others.” A more detailed composition of each PrimPol-PV1 family is provided in Supplementary data file 1. Taxonomic distribution of bacterial AEP of the PrimPol-PV1 supergroup. Taxa containing less than 4% (10% for RepB′) of representatives were merged into group “others.” A more detailed composition of each PrimPol-PV1 family is provided in Supplementary data file 1.

Almost all PrimPol-PV1 members (91%) are encoded by highly diverse bacteria belonging to 12 bacterial phyla and their viruses (Fig. 4; Supplementary data file 1). The PolpTN2-like family is the only AEP family that exclusively includes archaeal members. It contains proteins encoded by plasmids and integrated mobile genetic elements (MGEs) from six archaeal species belonging to the order Thermococcales (phylum Euryarchaeota) [21], [22], although euryarchaeal proteins (from orders Methanococcales and Archaeoglobales) are also found in the PrimQH family (Supplementary data file 1). In addition, the *Anabaena* all3500-like family contains a single protein sequence from an uncharacterized archaeon GW2011_AR11. Similarly, the PrimPol proper family is characterized by a mixed membership of proteins encoded by pRN1-like plasmids from the archaeal phylum Crenarchaeota and homologs from diverse bacteria.

Although not very numerous, viral AEP homologs appear to be more diverse than archaeal ones and are found in four out of seven AEP families of the PrimPol-PV1 supergroup (Fig. 4, Supplementary data file 1). The *Anabaena* all3500-like family includes the largest proportion (7%) of viral sequences (Fig. 4). Two AEP homologs were also annotated as being encoded by fungi; however, the corresponding genes are present on relatively small (19 and 40 kb) genomic contigs containing other typical bacterial gene and thus, in all likelihood, represent contaminating bacterial sequences.

Different AEP families of the PrimPol-PV1 supergroup display distinct taxonomic distribution patterns in bacteria. The most widespread are PrimQH, all3500, and RepE/RepS families, each found in seven or more different bacterial phyla. PriQH, PriHYQ, all3500, and RepB′ families are predominantly found in Proteobacteria, whereas most of the InversePrim members are encoded by Firmicutes (Fig. 4). It should be noted that these results are likely to be biased by the current composition of the bacterial genomic databases which are dominated by proteobacteria and firmicutes. This potential bias notwithstanding, some observations are noteworthy. Of particular interest is the apparent specificity of the RepE/RepS family to the gut-associated microbiota. More than half of the members represent sequences extracted from the gut metagenomes of various mammals (human, rat, pig, mouse) (Fig. 4). Consistently, additional quarter is derived from Chlamydiae, Bacteroidetes, Fusobacteria, and Firmicutes, all of which are prominent components of the gut microbiome. None of the other six PrimPol-PV1 families is found in Chlamydiae, Bacteroidetes, or Fusobacteria, emphasizing the uniqueness of the RepE/RepS family. Notably, many of the RepE/RepS proteins are encoded on extrachromosomal plasmids (Fig. S1), which might have an important impact on the gut microbiome, for example, by serving as vectors for transfer of antibiotic resistance genes or modulating conflicts in microbial populations via toxin/bacteriocin production and secretion.

### PrimPol-PV1 primases in bacterial genomes are encoded within integrated mobile genetic elements

**Fig. 5:**
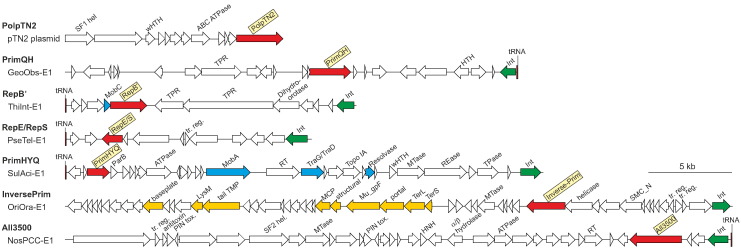
Selected genome maps representing the diversity of MGEs encoding AEPs from the seven families of the PrimPol-PV1 supergroup. Each family is represented by one genome map. The maps are drawn roughly to scale. The protein-coding genes are shown by arrows indicating the direction of transcription, whereas tRNA genes are depicted by red bars. The blank arrows show poorly conserved genes, many of them encoding uncharacterized proteins. AEPs from different families are shown in red; genes encoding mobilization proteins are in cyan; integrase genes (Int) are in green, and genes encoding proteins responsible for virion assembly are depicted in yellow. Abbreviations: SF1 and SF2 hel, helicases of superfamilies 1 and 2, respectively; wHTH, winged HTH; TPR, tetratricopeptide repeat-containing protein; tr. Reg., transcription regulator; RT, reverse transcriptase; Topo IA, topoisomerase IA; MTase, DNA methyltransferase; REase, restriction endonuclease; TPase, transposase; TMP, tape measure protein; MCP, major capsid protein; TerS and TerL, small and large subunits of the terminase complex, respectively; SMC_N, N-terminal ATPase domain of the structural maintenance of chromosomes protein; tox., toxin; HNH, HNH family nuclease.

Although some members of the PrimPol-PV1 supergroup are encoded by bacteriophages and plasmids, the provenance of the chromosomally encoded PrimPol-PV1 homologs (90% of proteins in our data set) remained unclear. Indeed, some of the AEP families are responsible for bona fide cellular functions as in the case of bacterial LigD complex responsible for the NHEJ pathway of double-strand break repair [7], [8] or human PrimPol which facilitates replication fork progression by acting as a translesion DNA polymerase or as a specific DNA primase reinitiating downstream of lesions that block DNA synthesis [17], [29]. Thus, to determine the potential function of PrimPol-PV1 primases in bacterial genomes, we performed comprehensive analysis of the genomic loci containing representative genes from all bacterial PrimPol-PV1 families. To obtain sufficient genomic context, for this analysis, we selected primases encoded within complete genomes or on large genomic contigs (> 30 kb). In all but one cases analyzed, we obtained unequivocal evidence that genes encoding PrimPol-PV1 primases are carried by integrated MGE. Furthermore, in 25 out of 31 genomes investigated, we could determine precise coordinates of MGE integration (Table S1). These MGE were surrounded by characteristic direct repeats corresponding to the attachment sites generated by integrases of the tyrosine or serine recombinase superfamilies. Most of the MGEs were integrated into various tRNA genes (*n* = 14) or intergenic regions (*n* = 9), whereas two elements were integrated into the 3′-distal region of the gene encoding for the GMP synthase (AEJ11636) and a tmRNA gene, respectively (Table S1). The detected elements have highly variable gene contents (Fig. 5) and could be grouped into three major classes: (i) prophages derived from members of the order *Caudovirales*; (ii) conjugative and mobilizable MGE, encoding conjugation apparatus or a mobilization protein required for initiation of DNA transfer, respectively; and (iii) cryptic integrated MGE lacking homologs of conjugation or phage genes. Interestingly, all analyzed elements encoding primases of the InversePrim family correspond to prophages, although out of 59 InversePrim proteins in our data set, only 2 are encoded by bona fide viral genomes (Supplementary data file 1). However, primases of other families are also occasionally encoded in prophages, whereas the PrimQH primase of the archaeaon *Methanoccocus voltae* A3 is encoded within a previously described provirus A3-VLP related to spindle-shaped archaeal viruses [30], [31]. Collectively, these results indicate that AEPs of the PrimPol-PV1 supergroup are (nearly) exclusive to MGE and in all likelihood play crucial role in their replication (hence the name of the supergroup). This finding also helps to rationalize the wide taxonomic distribution of AEPs in bacteria.

### Analysis of domain organizations within the PrimPol-PV1 supergroup reveals novel families of putative helicases

AEP domains are often fused to various helicases, DNA-binding modules, and/or α-helical domains known as PriCT-1 and PriCT-2, which have also been shown to bind to DNA [3], [32]. Analysis of domain organization can provide valuable information on the range of activities associated with a given protein. For instance, viral replicative primases are typically fused to helicase domain or are encoded in its vicinity [9]. Thus, to gain further insights into the diversity of the PrimPol-PV1 primases, we used sensitive profile-profile searches with HHsearch to systematically explore their domain organizations.

#### PolpTN2-like family

**Fig. 6:**
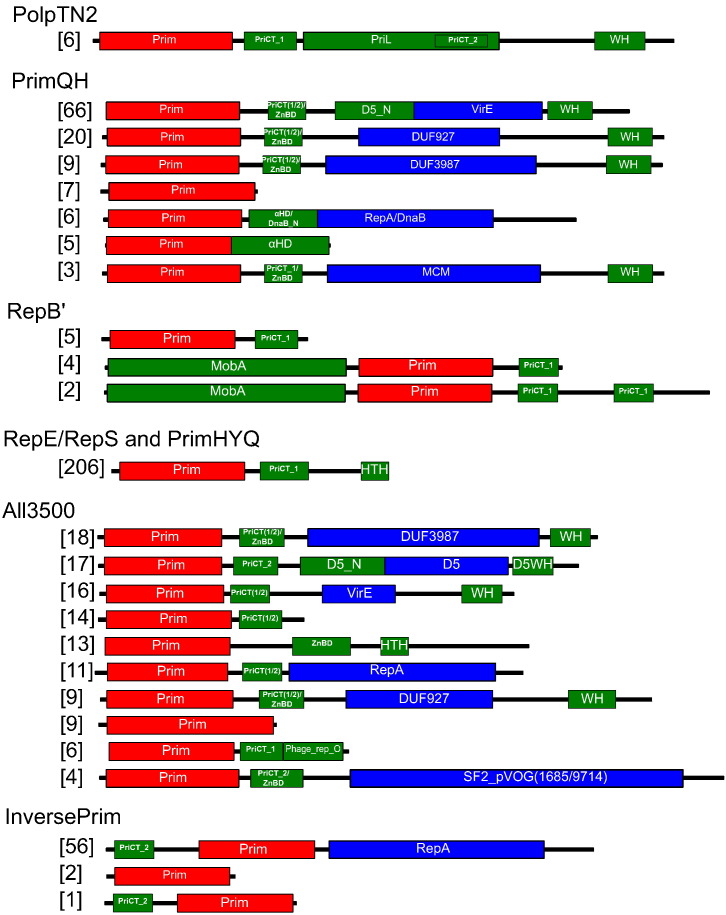
Diversity of domain organizations in the PrimPol-PV1 supergroup. The number of representative sequences having specific domain organization is indicated on the left. The catalytic helicase domains are shown in blue; primases, red; other domains, green. WH, winged HTH domain; αHD, α-helical domain; MCM, minichromosome maintenance helicase; SF2, superfamily 2 helicase.

Previous analysis has suggested that PolpTN2 is a fusion of two domains: an N-terminal AEP domain and a C-terminal domain homologous to the large subunit (PriL) of archaeal and eukaryotic primases [20]. Using more sensitive sequence analysis methods, we found that PolpTN2-like proteins in addition possess a PriCT-1 domain located between the AEP and PriL-like domains, whereas the C-terminal region following the PriL-like domain can be confidently (94% HHsearch probability) predicted to adopt a winged-helix (WH) domain (Fig. 6). To see how well sequence of PolpTN2 from *Thermococcus nautili* plasmid pTN2 would fit into a structure of a WH domain fold, we submitted it to two structure prediction servers, I-TASSER [33] and RaptorX [34]. The C-terminal WH domain of *Salmonella enterica* Typhi protein STY4665 (PDB: 2ipq), a relaxase belonging to the PFL_4751 family widespread in various bacterial integrative and conjugative elements [35], was identified as the best template for modeling. The resultant structural model was characterized by a good quality score (Table S2, Fig. S3), reinforcing the prediction that the C-terminal domain of PolpTN2 family proteins adopts a WH fold. In order to detect features common to structurally related WH domains, we aligned the corresponding domain of PolpTN2 with the closest structural homologs detected using the Dali server [36]. Sequence alignments and structure comparisons indicate that the WH domain of PolpTN2, similarly to the WH domain of the STY4665 relaxase and SCCmec helicase, but differently from the canonical WH domains, has a four-stranded β-sheet that forms an open β-barrel, instead of three-stranded or two-stranded β-sheets found in ORC/Cdc6 and MCM, respectively (Fig. S4B) [37]. Nevertheless, the WH domain of PolpTN2 shares with other WH domains a set of conserved aromatic amino acid residues which might be important for DNA binding or structure stabilization (Fig. S4A). Given the structural similarities, it is tempting to speculate that the C-terminal WH domain of PolpTN2 might be responsible for recognition of the origin of replication of the cognate plasmid (Fig. S4B). This function is likely to be mediated by the positively charged and aromatic residues present in the “recognition” helix (α3) and the “wing” region (Fig. S4).

#### PrimQH family

All except seven members of the PrimQH family are multidomain proteins (Fig. 6). In 84% of PrimQH members, the primase domain is followed by a PriCT-1/2 or Zn-binding domain (ZnBD), whereas the C-terminal region is occupied by the WH domain, which might be involved in origin recognition, like in the case of PolpTN2-like enzymes discussed above. Between the PriCT-1/2/ZnBD and the WH domains, the majority of proteins in this family contain diverse P-loop ATPase domains. Whereas some of them are related to known helicases, including RecA-type helicases of the RepA and DnaB families as well as the MCM-family helicase, many representatives contain experimentally uncharacterized AAA+ superfamily ATPase domains of the VirE family (PF05272) or two different domains of unknown function (DUF), namely, DUF927 and DUF3987 (Fig. 6). Given that these ATPase domains are associated with primases and substitute for known helicase domains (MCM, RepA, DnaB), we predict that they represent novel families of helicases involved in MGE replication (Fig. S5). Indeed, it has been recently demonstrated that DUF927 family protein encoded by mobile genomic island of *Staphylococcus aureus* is a bona fide helicase [38]. The VirE domain is present in more than half (56%) of the representatives. Notably, MCM helicases are generally considered to be specific for eukaryotes and archaea, where they are principal replicative helicases. In the bacterial realm, MCM-family helicases have been previously described only in bacteriophages [9], [39], [40]. Our current analysis suggests that MCM-like helicases are also encoded by bacterial (integrative) plasmids (Supplementary table S1), suggesting that MCM-encoding genes are more broadly distributed among bacterial MGE than previously recognized. In the case of archaeal MCM-encoding MGE, it has been demonstrated that the corresponding genes have been acquired from the respective hosts on several independent occasions [41]. A similar evolutionary scenario cannot be postulated for the *mcm* genes encoded by bacterial MGE, because bacteria do not employ MCM helicases in any of the steps of chromosomal replication, recombination, or repair. Furthermore, given that the bacterial MCM proteins are highly divergent, recent horizontal gene transfer from archaea appears unlikely.

#### RepB′ family

With 11 representatives, RepB′ family is the second smallest among PrimPol-PV1 families. Nevertheless, it displays three different patterns of domain organization (Fig. 6). The prototype of this family, RepB′ from plasmid RSF1010 [13], has a simple organization where the primase domain is followed by the PriCT-1 domain. However, in other members, the primase domain is preceded by a relaxase domain related to those found in nickases of the HUH superfamily and involved in plasmid mobilization through conjugation [42]. Interestingly, plasmid RSF1010 also encodes a related nickase (MobA) but as a separate gene [43]. Thus, the coupling of MobA-like and primase domains is unlikely to play a direct role in plasmid replication, but rather might assist in orchestration of the plasmid replication and conjugation. MobA domain-containing proteins also possess one or two PriCT-1 domains at the C-terminus.

#### RepE/RepS and PrimHYQ families

RepE/RepS and PrimHYQ families collectively have more members than any other PrimPol-PV1 family. Nevertheless, all representatives of the two families have a uniform domain organization: PriCT-1 and HTH domains are fused to the C-terminus of the primase domain (Fig. 6, Supplementary data file 1). Remarkably, not only do the primases of the two families lack putative helicase domains, but the putative replicative helicase genes are also not present in the corresponding MGE (Table S3), raising questions as to how the MGE DNA is unwound during the replication. This is surprising in the light of our previous observation that nearly all known dsDNA viruses encoding primases also encode a replicative helicase, with only about 2% deviating from this pattern [9]. *Bacillus* phage IEBH, encoding a primase of the PrimHYQ family, represents one of such rare cases. Recent studies performed with the primase of the ColE2 family [3], which is distantly related to the RepE/RepS family and has the same domain organization, might hold a clue to this conundrum. It has been demonstrated that although ColE2 Rep lacks the NTPase domain, it still can unwind dsDNA using the PriCT-1 and HTH domains fused to the primase domain [32]. Thus, we predict that the PriCT-1-HTH module consistently conserved in members of the RepE/RepS and PrimHYQ families also has the DNA unwinding activity.

#### All3500 family

All3500 family displays the highest variation of domain organizations in the PrimPol-PV1 supergroup (Fig. 6). Some all3500-like proteins possess similar domain organizations to those observed in the PrimQH family, with the primase domain fused to various (putative) helicase domains, including RepA, DUF927, DUF3987, and VirE. A large number of proteins (*n* = 17) contains a domain homologous to the superfamily 3 helicase D5 of vaccinia virus, which is conserved in a range of eukaryotic viruses with large and giant genomes [44]. Another notable group of proteins has C-terminal domains which show only remote similarity to helicases of the superfamily 2. Their closest homologs are found in some T4-like phages and *Bacillus* phage Spbeta. These uncharacterized phage proteins have been classified into prokaryotic virus orthologous groups (pVOG) 1685 and 9714 [45]. Interestingly, members of the pVOG9714 are most similar to the origin-binding protein (UL9) of eukaryotic herpesviruses (Pfam id: PF02399). We predict that pVOGs 1685 and 9714 represent a novel family of helicases specific to viruses and plasmids (Fig. S5). Finally, six proteins correspond to a unique fusion of the primase domain with the PriCT-1 and a domain homologous to the replication protein O of bacteriophage λ (Pfam id: PF04492). Protein O initiates replication of bacteriophage DNA by interacting with the replication origin [46]. Conceivably, the C-terminal O-like domain recruits the primase to the replication origin, where it initiates the primer synthesis.

#### InversePrim family

Similar to members of RepE/RepS and PrimHYQ families, the overwhelming majority of representatives (95%, *n* = 56) of the InversePrim family have the same domain organization (Fig. 6). The primase domain is fused to the RepA family helicase and the PriCT-1 domain. However, unlike in all other PrimPol-PV1 families, the PriCT-1 domain does not follow the primase domain, but rather precedes it (hence the family name).

### PriCT and Fe-S domain of primase large subunit are related

**Fig. 7:**
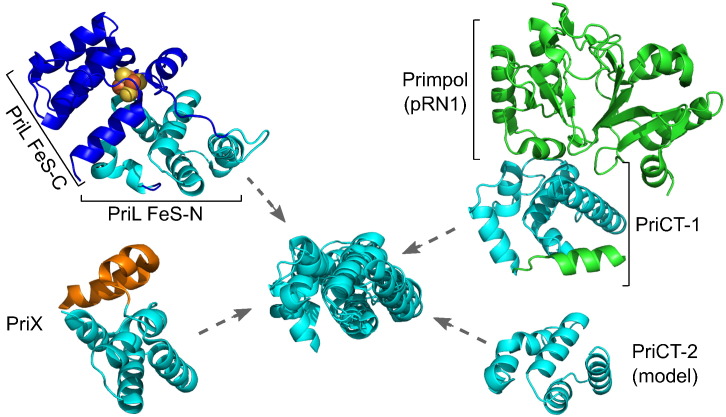
Superimposition of pRN1 Primpol PriCT-1 domain (PDB: 3m1m), PriCT-2 domain of *B. badius* protein (WP_063441057), PriX of *Sulfolobus solfataricus* (PDB: 4wyh) and the N-terminal part (PriL Fe-S_N) of *Saccharomyces cerevisiae* primase large subunit Fe-S domain (PDB: 3lgb). Atoms of Fe-S cluster are shown as spheres.

The strong coupling of either PriCT-1 or PriCT-2 domain with the primase domain strongly suggests that the two domains are functionally interchangeable and play a profound role during primer synthesis. It has been previously noted that the “α-helical subdomain” of Primpol from plasmid pRN1, which we identify here as PriCT-1 domain, bears structural relatedness to the C-terminal part of the PriL [47]. The C-terminal domain of the PriL is composed of a larger (Fe-S_N) and a smaller (Fe-S_C) α-helical subdomains with Fe-S cluster at their interface [6]. The structure of the PriCT-2 domain is unknown; however, it has been previously hypothesized that PriCT-1 and PriCT-2 might be distantly related [3]. Unfortunately, at the time, this hypothetical relationship could not be substantiated. To further explore the provenance of the PriCT domains, we performed both sequence profile-based and structure-based analysis of PriCT domains. When the PriCT-2 sequence profile from *Bacillus badius* (WP_063441057) was used as a query, HHsearch found Fe-S domain of archaeal PriL (CDD: PRK02249) with high probability of 95% and aligned two of its four cysteine residues (Fig. S6). Structural modeling and structure similarity search using Dali server have further supported the PriCT-2 structural similarity to the larger domain of the PriL C-terminal region (Fig. 7; Tables S2 and S4). Taken together these observations suggest that PriCT-1 and PriCT-2 are related to each other and to PriL Fe-S_N. Moreover, all three of them are structurally similar (Table S4, Fig. 7) to PriX, the recently identified non-catalytic subunit of the archaeal primases essential for efficient primer synthesis [48]. Thus, we hypothesize that both PriCT domains are functional counterparts and bona fide homologs of the PriL subunit of archaeal and eukaryotic primases. Whereas PriL in cellular primases is usually encoded by a separate gene, the PriCT domains are most commonly fused to the MGE-encoded primases.

### Concluding Remarks and Evolutionary Considerations

The diversity and abundance of AEP homologs in bacteria is rather astounding, especially considering that AEPs belong to the core DNA replication machinery of archaea and eukaryotes, but not bacteria, which instead use DnaG family primases. In this study, using an archaeal primase–polymerase PolpTN2 as a seed for sequence similarity searches, we recovered over 800 AEP homologs in bacteria from 12 highly diverse phyla, including not only those overrepresented in genomic databases, such as Proteobacteria, Firmicutes, and Actinobacteria, but also those from less sampled phyla, such as Deinococcus–Thermus, Fusobacteria, and Verrucomicrobia. Notably, for our searches, we used a protein database that was filtered to 70% identity (i.e., all sequences that were more than 70% identical to any other sequence in the database were removed), suggesting that the actual number of PolpTN2-like bacterial AEP sequences is considerably greater. Clustering and community detection analyses have uncovered five novel AEP families, which together with two previously defined families form a large supergroup, dubbed PrimPol-PV1. The families displayed non-uniform taxonomic distribution, with one family apparently being specific to animal gut-associated microbiota. The defining feature of the PrimPol-PV1 supergroup is the conservation of an arginine residue in the newly defined motif IV located within the flange region as well as the relaxed conservation of the active site histidine residue in motif II, which was not previously reported in other AEP families [3]. Structural modeling of the PolpTN2 AEP domain suggests that the two residues, although separated in the primary protein structure, are in proximity in the three-dimensional structure and likely play a similar role in positioning of the substrate nucleotides. Moreover, covariation analysis revealed a third residue that coevolves with the latter two, and thus, the three residues are likely to be functionally connected. Systematic dissection of the domain organizations in the PrimPol-PV1 supergroup showed that in most of the proteins, the AEP domain is fused to various other functional modules, among which the α-helical PriCT-1 and PriCT-2 domains are most common. Our results indicate that the two domains are homologous to each other and also to the C-terminal part of the cellular PriL subunit, revealing a previously overlooked evolutionary link between these functional modules. Notably, whereas the PriL is nearly exclusively found in association with DNA primases responsible for chromosomal replication, PriCT domains are exclusive to AEP encoded by MGE. Indeed, genomic neighborhood analysis provided strong evidence that nearly all PrimPol-PV1 members are encoded by highly diverse MGE, including viruses and plasmids. Unfortunately, due to high sequence divergence, it is not possible to predict with any certainty the vector of evolution between the PriL and PriCT domains, although the diversity as well as wide spread of the PriCT domains indicates that its association with AEP is likely to be ancient. Of particular interest is the provenance of AEP in bacterial MGE. Some replication-associated proteins have been recruited by MGE from their respective hosts, as is the case for replicative MCM helicases in archaea [41] or eukaryotic replication protein A [49]. However, given that bacterial PrimPol-PV1 members are more similar to the archaeal ones, the possibility that they were recruited by MGE from AEP domains of bacterial LigD appears unlikely. Consistently, PrimPol-PV1 proteins do not show appreciable sequence similarity to the AEP domain of LigD that lack the characteristic arginine residue in the flange region [3]. Instead, the near ubiquity of AEP domains in various MGE from all three domains of life suggests that this module has been recruited by MGE in a distant past, possibly prior to the divergence of modern cellular organisms from the last universal cellular ancestor, LUCA. This is in agreement with evolutionary scenarios in which MGE originated and diversified in an ancient viral world that predated LUCA [50], [51]. The widespread distribution of different AEP families in the three cellular domains and the corresponding viruses and plasmids suggests that these families had possibly already diverged at the time of LUCA. However, the diversity and abundance of PrimPol-PV1 members in Bacteria, their scarcity in Archaea, and their apparent absence in Eukarya suggest that this particular AEP supergroup originated and diversified in MGE coevolving with the cellular lineages leading from LUCA to the LBCA (The Last Bacterial Common Ancestor). In that case, the archaea-specific PolpTN2 family could have been horizontally transferred from Bacteria to Archaea via a promiscuous MGE. However, considering that the PolpTN2 family is not specifically related to any of the various bacterial PrimPol-PV1 families, one cannot exclude that MGEs encoding the ancestor of PrimPol-PV1 proteins were already around at the time of LUCA. Further exploration of the MGE world in Archaea should help to discriminate between these hypotheses. In the course of evolution, the AEP domain has been fused on multiple occasions with various functionally coupled domains, such as helicases or DNA recognition and binding modules. This is well illustrated by our observation that some of the AEP domains of the PrimPol-PV1 supergroup are fused to experimentally uncharacterized candidate helicases. Beyond doubt, a systematic search for domains coupled with various primases and helicases should result in identification of novel enzymes/domains involved in DNA replication, recombination, and repair, which might have unexpected enzymatic activities for potential biotechnological applications.

## Materials and Methods

### Databases

Protein sequence databases were obtained from NCBI† and UniProt‡. NCBI “nr” and UniProt databases were filtered to 70% and 50% identity, respectively (nr70 and uniref50). Sequence profile database included profiles from PDB§, SCOP [52], Pfam [53], and pVOGs [45] databases.

### Sequence searches and clustering

Initially, the primase domain of PolpTN2 (YP_003603593.1, 1–257 aa) was used as a query to search for homologs in the nr70 database with Jackhmmer [54]. After seven Jackhmmer iterations, significantly (*E*-value ≤ 1e − 03) matching sequences were extracted and grouped using CLANS [24]. Major groups of PolpTN2 primase domain homologs were then identified using CLANS convex clustering algorithm at *P*-value = 1e − 09. Next, sequences from the resulting groups were extracted and merged with homologs of well-characterized members of the AEP superfamily. The latter sequences were retrieved from uniref50 database using seven Jackhmmer iterations. All the merged sequences were then again grouped using CLANS. The list of PrimPol-PV1 sequences used for CLANS clustering is available for download (Supplementary data file 3). Sequence logos for each group were produced using the WebLogo server [55].

### Domain identification

To identify the primase domain, sequences in each group of PolpTN2 homologs were aligned using MAFFT [56] optimized for accurate local alignment (option “L-INS-i”). Next, sequence regions longer than 50 residues on either side of the primase domain were extracted for domain identification. For every such region, we generated sequence profile by running two iterations of Jackhmmer [54] against the nr70 sequence database (the NCBI “nr” database filtered to 70% identity) using *E*-value = 1e − 03 or a more stringent inclusion threshold. The resulting profiles were used to search against profile databases with HHsearch [57].

### Homology modeling

Protein structures were modeled by submitting corresponding protein sequences to automatic homology modeling servers, RaptorX [34] and I-TASSER [33]. Two different servers were used to ensure the reliability of obtained models following the observation that, if models generated by different methods are similar, these models can be expected to be reliable. In all cases, servers returned similar models having identical fold. In addition, models were evaluated with ProSA-web [58] (Table S2). Top scoring models were used for further analyses. Structures were aligned using either Dali [59] or the “cealign” command in PyMol¶.

### Analysis of residue coevolution

Covariation analysis was performed using PSICOV [60] and PLMC script from EVmutation package [61]. Initial sequence alignment was generated by Jackhmmer after 10 iterations against nr70 database using PolPTN2 primase domain as a query. Sequences containing gaps in positions 179 or 231 were removed from the alignment. Final alignment used by PSICOV and PLMC had 1427 sequences. Covariation scores are available for download (Supplementary data file 2).

### Identification and annotation of integrated MGEs

The integrated MGEs were identified by thorough analysis of genomic neighborhoods of the AEP-encoding genes. The precise borders of integration were defined based on the presence of direct repeats corresponding to attachment sites. The repeats were searched for using Unipro UGENE [62]. Genes of integrated MGE were annotated based on the PSI-BLAST searches [63] against the non-redundant protein database at NCBI and HHpred searches [57].

### Mutagenesis, purification, and activity assays with the PolpTN2 variants

The M1 in which the two catalytic Asps of the motif I were changed to Leu residues (DID → LIL) as well as the R231A mutant in which the Arg231 was substituted for an Ala were constructed by site-directed mutagenesis using the QuickChange II kit (Agilent), according to the manufacturer's instructions. The wt and mutant proteins were expressed at 37 °C using the *E. coli* Rosetta (DE3) pLysS strain (Novagen) and the 2xYT medium (BIO 101 Inc.). When the cell culture reached an OD600 nm of 0.8, induction at 15 °C was performed overnight with 0.5 mM IPTG (Sigma). Cells were harvested by centrifugation and resuspended in buffer A [20 mM Tris–HCl (pH 7.5), 200 mM NaCl, 5 mM β-mercaptoethanol]. Cell lysis was completed by sonication and the lysate was heated for 20 min at 70 °C before centrifugation at 20,000 rpm for 20 min. The soluble fraction was loaded on a Ni-NTA column (Qiagen Inc.) equilibrated with buffer A. The protein was eluted with imidazole and subsequently loaded on a heparin column (GE Healthcare) equilibrated against buffer A′ [20 mM Tris Tris–HCl (pH 7.5), 50 mM NaCl, 5 mM β-mercaptoethanol]. Elution was performed using a gradient between buffers A′ and B [20 mM Tris Tris–HCl (pH 7.5), 1 M NaCl, 5 mM β-mercaptoethanol]. The proteins were eluted at ∼ 0.9 M NaCl. Eluted fractions were pooled and loaded on a Superdex200 column (Amersham Pharmacia Biotech) equilibrated against buffer A supplemented with 10 mM β-mercaptoethanol. The homogeneity of the protein samples was checked by SDS-PAGE. The DNA polymerase and DNA primase assays were performed following the protocols described by Soler *et al*. [22] and Gill *et al*. [20], respectively, with no modifications. For the primases assay, the 3′ end of the template oligonucleotide was blocked with a spacer C3 phosphoramidite (Sigma-Aldrich), a three-carbon spacer that efficiently blocks extension of the oligonucleotide by polymerase or terminal transferase.

The following are the supplementary data related to this article.

Supplementary Tables S1-S4 and Figures S1-S6 Image 2

Supplementary data file 1 Taxonomic distribution and domain organizations of PrimPol-PV1. Supplementary data file 1

Supplementary data file 2 Covariation of residues in the PrimPol domain of PrimPol-PV1 and PriS homologs. Supplementary data file 2

Supplementary data file 3 PrimPol-PV1 sequences used for CLANS clustering. Supplementary data file 3

## Footnotes

† ftp://ftp.ncbi.nlm.nih.gov/blast/db/.

‡ http://www.uniprot.org/downloads/.

§ www.pdb.org.

ǁ https://www.pymol.org/
