# Supplementary material for: Novel Families of Archaeo-Eukaryotic Primases Associated with Mobile Genetic Elements of Bacteria and Archaea
Source: J Mol Biol. 2018 Mar 2;430(5):737–50. doi: 10.1016/j.jmb.2017.11.014 (PMC5862659; doi:10.1016/j.jmb.2017.11.014)
Supplement: Supplementary file 1 — Supplementary Tables S1-S4 and Figures S1-S6 [file mmc1.pdf]

SUPPLEMENTARY TABLES AND FIGURES

Novel families of archaeo-eukaryotic primases in mobile genetic elements of Bacteria and Archaea

Darius Kazlauskas[1], Guennadi Sezonov[2], Nicole Charpin[3], Česlovas Venclovas[1]*, Patrick Forterre[3], Mart Krupovic[3]*

[1] – Institute of Biotechnology, Vilnius University, Saulėtekio av. 7, Vilnius 10257, Lithuania.
[2] – Sorbonne Universités, UPMC Université Paris 06, CNRS, UMR 7138 Evolution Paris Seine - Institut de Biologie Paris Seine, Paris 75005, France.
[3] – Unité Biologie Moléculaire du Gène chez les Extrêmophiles, Department of Microbiology, Institut Pasteur, 25 rue du Docteur Roux, Paris 75015, France.

* – Correspondence:
Mart Krupovic
Department of Microbiology, Institut Pasteur,
25 rue du Docteur Roux,
Paris 75015, France
Tel: +33 1 40 61 37 22
Fax: +33 1 45 68 88 34
E-mail: krupovic@pasteur.fr

Česlovas Venclovas
Institute of Biotechnology,
Vilnius University, Saulėtekio av. 7,
Vilnius 10257, Lithuania
Tel: +370 5 223 4368
Fax : +370 5 223 4367
E-mail: ceslovas.venclovas@bti.vu.lt

Table S1. AEP-encoding mobile genetic elements integrated into bacterial genomes.

Organism/Source	MGE	Accession #	Coordinates	Size, bp	Integration target	att length	Prim-pol accession
**InversePrim**							
Faecalibaculum rodentium Alo17	FqeRod-E1 (prophage)	CP011391	1141989..1187164	45,176	intergenic	17	AMK54645
Rummeliibacillus stabekisii PP9	RumSta-E1 (prophage)	CP014806	574484..627821	53,338	intergenic	31	AMW98431
Coriobacteriaceae bacterium CHKCI002	prophage	FCNB01000006					CVH74801
Lactobacillus acidipiscis DSM 15353	LacAci-E1 (prophage)	NZ_JQBK01000001	5759..46664	40,906	intergenic	16	WP_010494440
Oribacterium sp. oral taxon 078	OriOra-E1 (prophage)	NZ_GG729935	422961..462816	39,856	tmRNA	81	WP_009215761
Enterococcus cecorum strain G-29 29-G	EntCec-E1 (prophage)	NZ_LDEC01000022	71588..105903	34,316	intergenic	13	WP_047342731
**All3500**							
Anabaena variabilis ATCC 29413	AnaVar-E1 (iMGE, immobilized)	CP000117	3175655..3185901	10,247	tRNA-Cys	43	ABA22184
Rhodanobacter denitrificans 2APBS1	RhoDen-E1 (prophage remnant)	CP003470	1772768..1782398	9,631	tRNA-Leu	57	AGG88763
Nostoc sp. PCC 7120	NosPCC-E1 (iMGE)	NC_003272	4180299..4220107	39,809	tRNA-Gly	71	WP_010997650
Candidatus Rubidus massiliensis	RubMas-E1 (iMGE)	CCSC01000001	859355..872473	13,119	tRNA-Met	51	CDZ80274
Defluviimonas alba cai42	DefAlb-E1 (iMGE)	CP012661	1774090..1784184	10,095	tRNA-Ser	17	AMY69057
Aneurinibacillus soli	-	AP017312					BAU26382
**PrimHYQ**							
Rhodopseudomonas palustris BisA53	RhoPal-E1 (iMGE)	CP000463	609203..635904	26,702	tRNA-Ser	20	ABJ04522
Pseudomonas putida S16	PsePut-E1 (iMGE, conjugative)	CP002870	1183091..1198969	15,879	GMP synthase (AEJ11636)	16	AEJ11641
Sulfobacillus acidophilus TPY	SulAci-E1 (iMGE, conjugative)	CP002901	395154..423677	28,524	tRNA-Arg	59	AEJ38610
Faecalibaculum rodentium Alo17	FaeRod-E1	CP011391	540173..632467	92,295	intergenic	14	AMK53794
Achromobacter sp. 2789STDY5608633	iMGE, conjugative	CYTR01000011	end of contig				CUJ51042
Acetobacter cibinongensis 4H-1	AceCib-E1 (iMGE)	BAMV01000007	157045..193382	36,338s	intergenic	26	GAN59754
**PrimQH**							
Geodermatophilus obscurus DSM 43160	GeoObs-E1 (iMGE)	CP001867	3659031..3686057	27,027	tRNA-Val	53	ADB76084
Rhodoplanes sp. Z2-YC6860	iMGE (several integrase genes)	CP007440	?				AMN40183
Desulfovibrio magneticus RS-1	DesMag-E1 (iMGE)	AP010904	3398082..3430367	32,287	tRNA-Arg	46	BAH76511
Dethiobacter alkaliphilus AHT 1	iMGE (several integrase genes)	ACJM01000003	?				EEG78369
Tsukamurella pulmonis	TsuPul-E1 (iMGE)	LSRH01000040	419691..432504	12,814	tRNA-Met	46	KXO87840
Meiothermus silvanus DSM 9946	MeiSil-E1 (prophage)	NC_014212	1170155..1209571	39,417	tRNA-Thr	45	WP_013157668
Paenibacillus naphthalenovorans 32O-Y	PaeNap-E1 (iMGE)	NZ_CP013652	5084627..5102316	17,690	intergenic	23	WP_062410722
**RepE/RepS**							
Pseudoalteromonas telluritireducens DSM 16098 c30	PseTel-E1	LVCM01000024	12662..27356	14,694	tRNA-Pro	51	KYL32719
Methylobacterium sp. GXS13	MetGXS-E1	NZ_LKKO01000001	83715..116382	32,667	tRNA-Gly (tandem iMGE)	45	WP_058190088
Mitsuokella multacida DSM 20544	MitGXS-E1	ABWK02000012	26157..39864	13,708	intergenic	11	EEX69002
Clostridium sp. M62/1	iMGE, mobilizable	ACFX02000057	Short contig				EFE10682
**RepB'**							
Thiomonas intermedia K12	ThiInt-E1 (iMGE, mobilizable)	CP002021	2347248..2364630	17,383	tRNA-Thr	49	ADG31552
Scytonema tolypothrichoides VB-61278	ScyTol-E1	NZ_JXCA01000025	1819..53443	51,625	intergenic	12	WP_048872245

Table S2. ProSA-web scores of models and their templates.

Protein (domain)	Sequence accession and coordinates	Template(s) PDB code and chain	Template(s) ProSA-web score	Model ProSA-web score
PolpTN2 (primase)	YP_003603593.1:5-267	1g71_A, 1v34_A, 1zt2_A, 2faq_A, 2irx_A, 3h25_A, 4bpu_A 4mm2_A	[-6.03:-8.92]	-5.07
PolpTN2 (Winged-helix)	YP_003603593.1:791-892	2ipq_X	-4.69	-4.38
PrimQH_Bbadius (PriCT-2)	WP_063441057.1:389-466	1dnp_B, 1owo_A, 2xry_A, 3umv_B, 4rr2_D	[-4.55:-5.29]	-5.36

Table S3. Mobile genetic elements of RepE/RepS family.

Organism/Source	Plasmid/Element	Accession number	Coordinates	Integration target	att length	Prim-pol accession	Replicative helicase in a vicinity of primase domain
Pseudoalteromonas telluritireducens DSM 16098 c30	PseTel-E1	LVCM01000024	27872..42566	tRNA-Pro	51	KYL32719	-
Methylobacterium sp. Leaf465		NZ_LMRA01000010	~ 39647..49633			WP_056380176	-
Clostridium botulinum strain Colworth BL151	pBL151	KJ776577				AIW54528	-
Methylobacterium sp. GXS13	MetGXS-E1	NZ_LKKO01000001	83715..116382	tRNA-Gly (tandem el)	45	WP_058190088	-
Mitsuokella multacida DSM 20544	MitGXS-E1	ABWK02000012	26157..39864	intergenic	11	EEX69002	-
Uncultured prokaryote from Rat gut metagenome	pRGRH0358	LN853021				CRY94786.1	-
Uncultured bacterium from human gut	pTRACA10	AM263037				CAK18990	-
Uncultured prokaryote from Rat gut metagenome	pRGRH0110	LN852800				CRY93933	-
Uncultured bacterium from human gut	pTRACA22	FN429767				CAZ72208	-
Uncultured prokaryote from Rat gut metagenome	pRGFK1342	LN853905				CRY96985	-
Rattus norvegicus cecum metamobilome	pRGI00580	HG796345				CDL66141	-
Uncultured prokaryote from Rat gut metagenome	pRGFK1350	LN853912				CRY97015	-
Uncultured prokaryote from Rat gut metagenome	pRGRH0676	LN853296				CRY95579	-
Uncultured prokaryote from Rat gut metagenome	pRGRH0268	LN852940				CRY94512	-
Uncultured prokaryote from Rat gut metagenome	pRGFK1338	LN853901				CRY96969	-
Uncultured prokaryote from Rat gut metagenome	pRGRH0393	LN853043				CRY94871	-
Uncultured bacterium from human gut	pTRACA20	FN429766				CAZ72200	-
Uncultured prokaryote from Rat gut metagenome	pRGFK1700	LN854203				CRY97700	-
Uncultured prokaryote from Rat gut metagenome	pRGFK0888	LN853490				CRY96048	-
Plaque samples from 50 patients presenting with periodontal diseases	pTRACA41	HM560024				AEJ88191	-
Campylobacter coli	pCC31	NC_006134				YP_063400	-

Table S4. Results of a structural similarity search using Dali server and pRN1 Primpol PriCT-1 domain (PDB: 3m1m) as a query. Structures with longest alignments (five α helices) are shown in yellow background.

No.	Chain	Z-score	RMSD	Alignment length	Number of residues	Identity (%)	Molecule
1	3m1m-A	25.2	0.0	109	320	100	ORF904
2	1w36-F	5.1	3.0	64	1077	3	DNA HAIRPIN
3	3tvs-A	5.0	3.4	85	538	8	CRYPTOCHROME-1
4	3lgb-B	4.5	3.3	78	193	18	DNA PRIMASE LARGE SUBUNIT
5	1f5n-A	4.5	4.5	73	570	3	INTERFERON-INDUCED GUANYLATE-BINDING PROTEIN 1
6	1dg3-A	4.4	4.5	72	540	1	PROTEIN (INTERFERON-INDUCED GUANYLATE-BINDING
7	4wyh-A	4.4	2.5	64	99	13	PriX
8	1dnp-A	4.3	3.3	85	470	7	DNA PHOTOLYASE

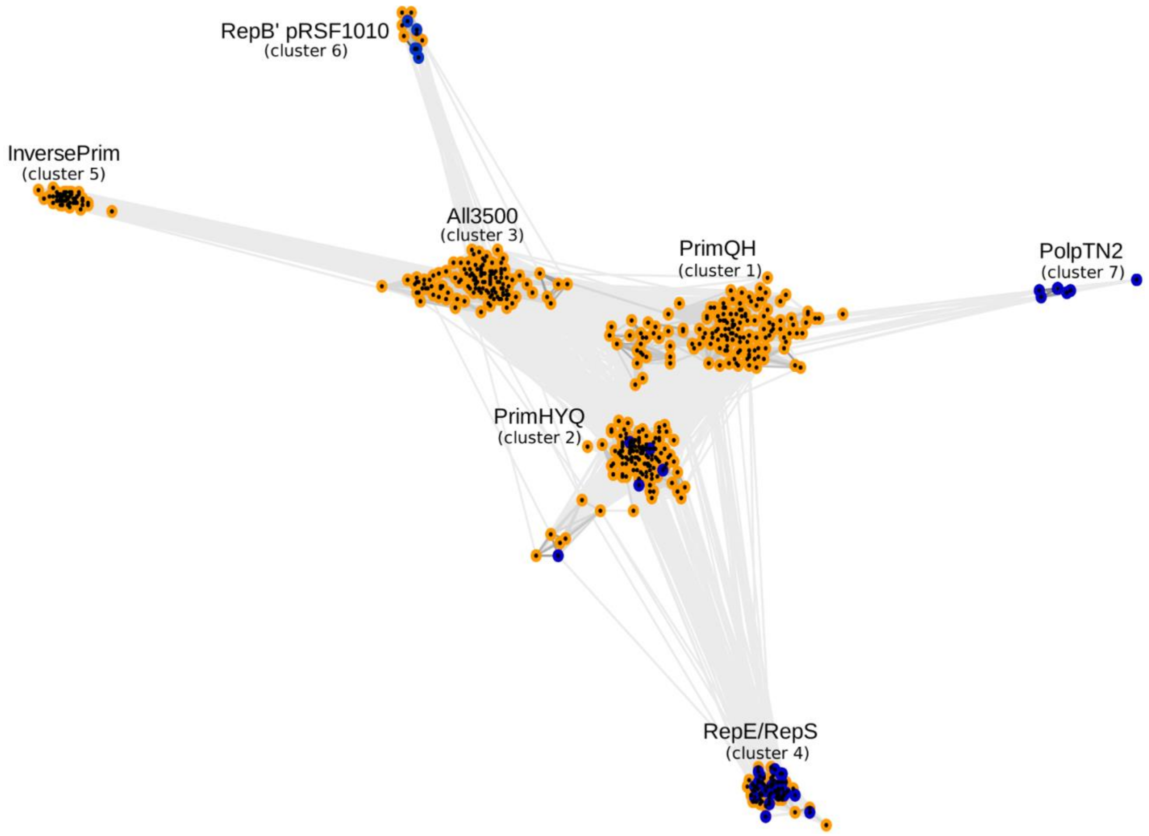

Figure S1. Diversity of PrimPol-PV1 proteins. Protein sequences were clustered by the pairwise sequence similarity (CLANS P-value ≤ 1e-09). PrimPol-PV1 proteins are shown as colored circles; blue color denotes sequences from plasmids.

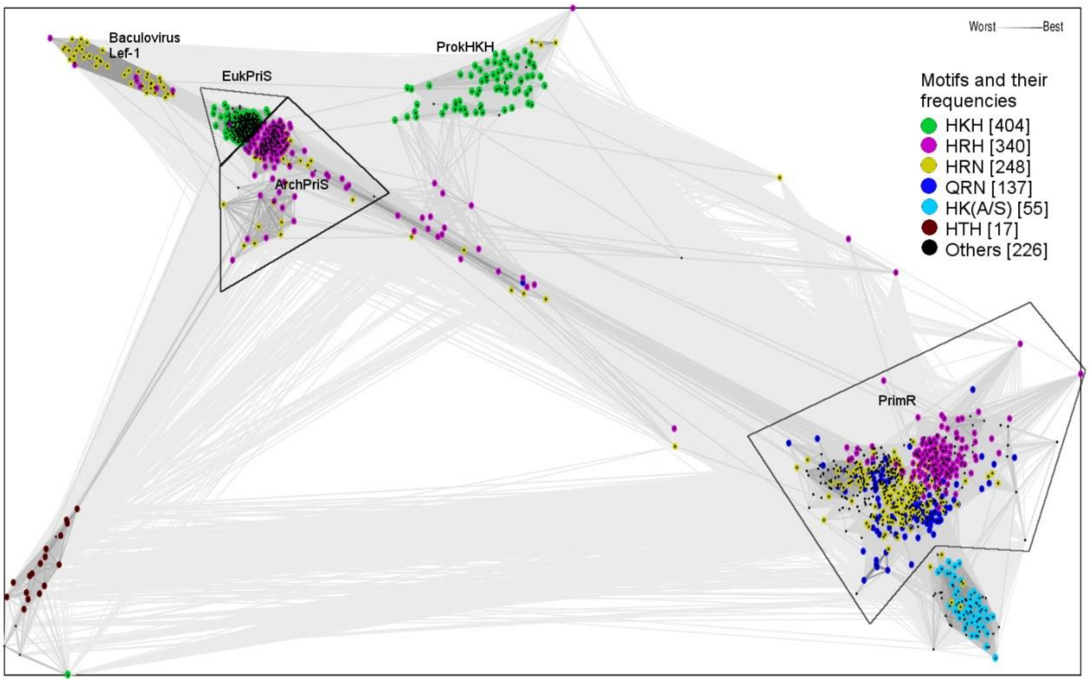

Figure S2. PolpTN2 primase domain homologs grouped by their pairwise sequence similarity (CLANS P-value ≤ 1e-05). Sequences are represented by circles which are colored according to motifs in a putative NTP-binding site.

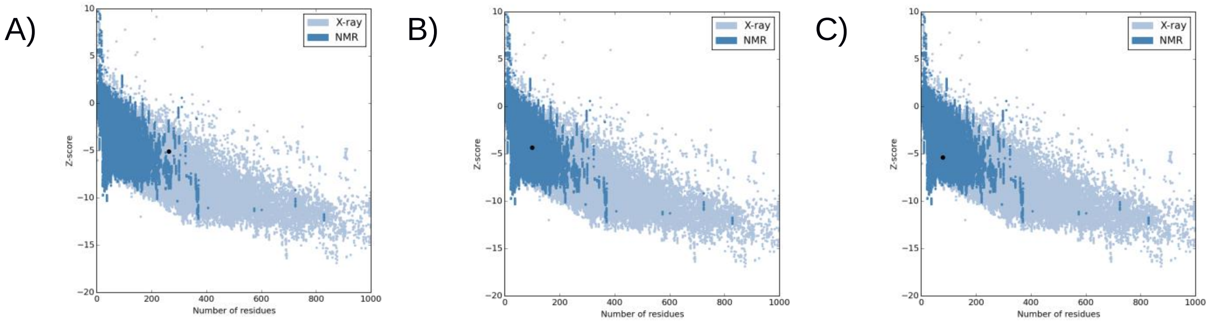

Figure S3. ProSA-web scores of protein structure models compared to scores of experimentally determined structures. (A) Primase domain (PolpTN2; Z=-5.07). (B) Winged-helix (PolpTN2; Z=-4.38). (C) PriCT-2 (PrimQH_Bbadius; Z=-5.36).

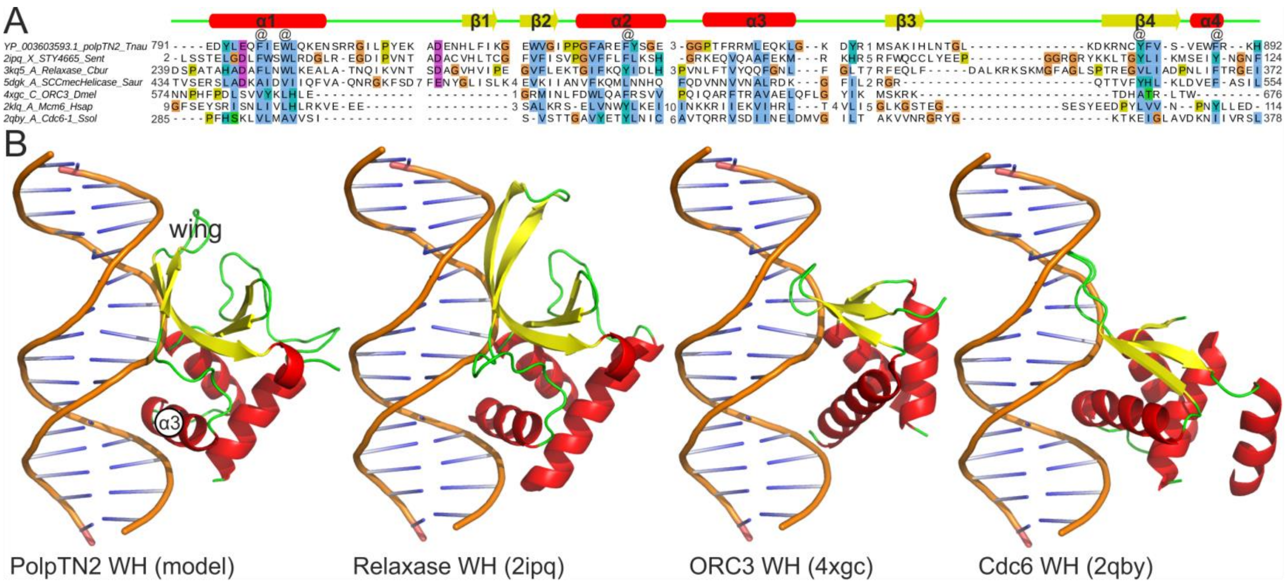

Figure S4. Comparison of winged-helix (WH) domain proteins. A. Structure-based alignment of DNA-binding WH domains from diverse proteins. Secondary structure of PolpTN2 shown above the alignment was predicted by RaptorX server. Numbers inside the alignment indicate number of omitted residues; @, conserved aromatic amino acid residues. Species abbreviations: Tnau, *Thermococcus nautili*; Sent, *Salmonella Enterica*; Cbur, *Coxiella burnetii*; Saur, *Staphylococcus aureus*; Dmel, *Drosophila melanogaster*; Hsap, *Homo sapiens*. B. Structures of WH domains aligned to a complex of WH domain and DNA from Cdc6 (PDB: 2qby). Molecules are colored by secondary structure (α helices, red; β sheets, yellow; random coils, green).

A)

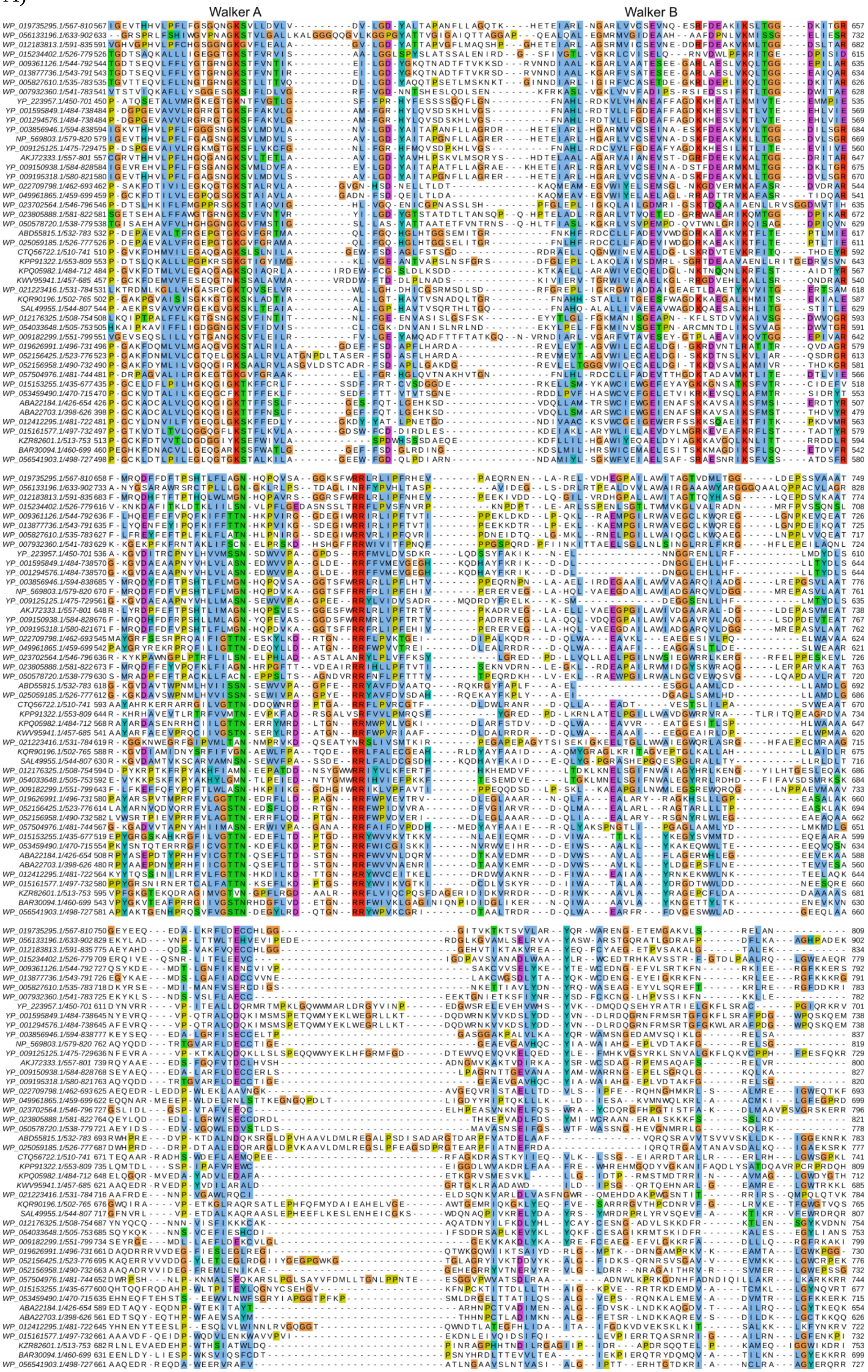

B)

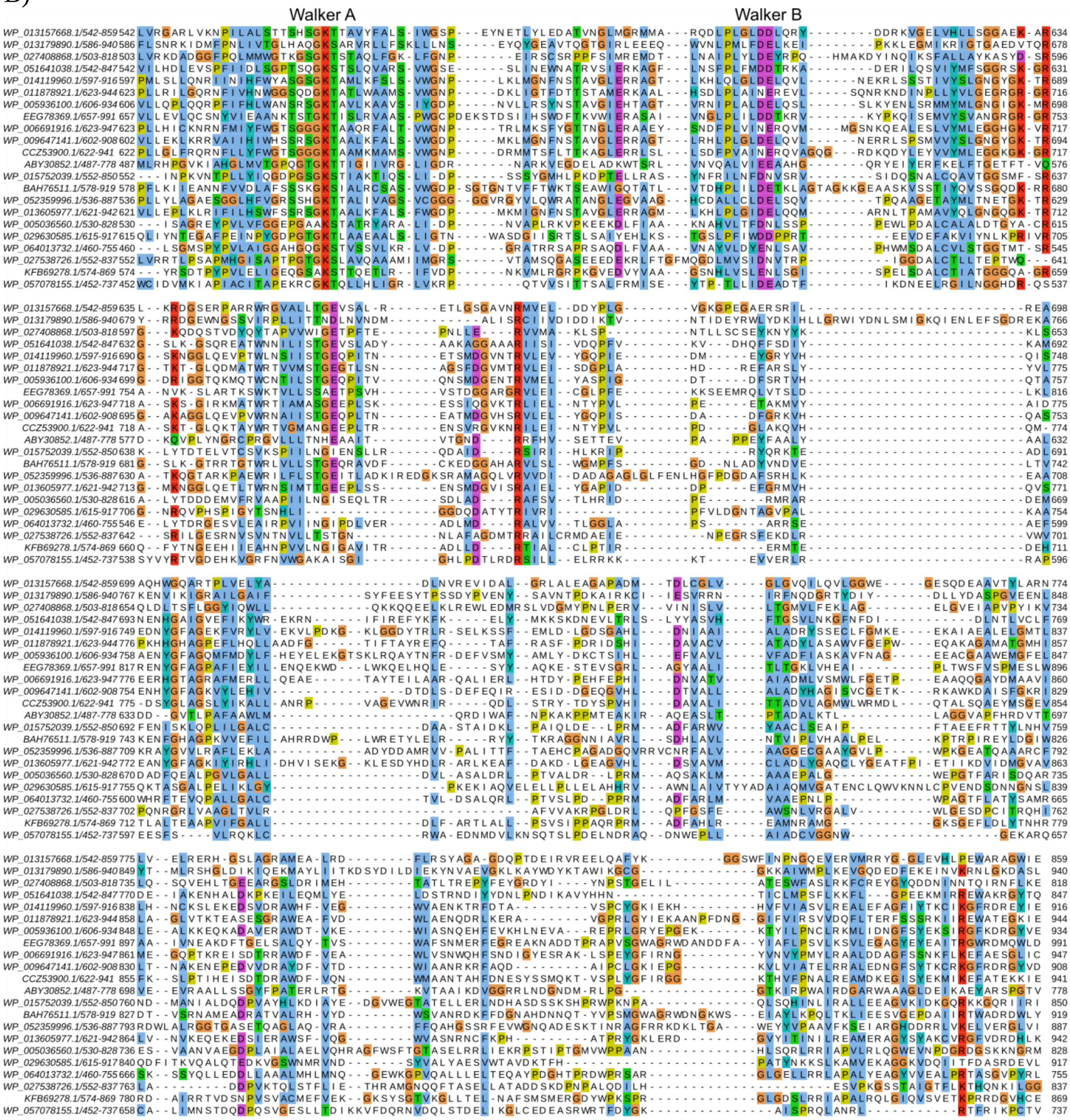

C)

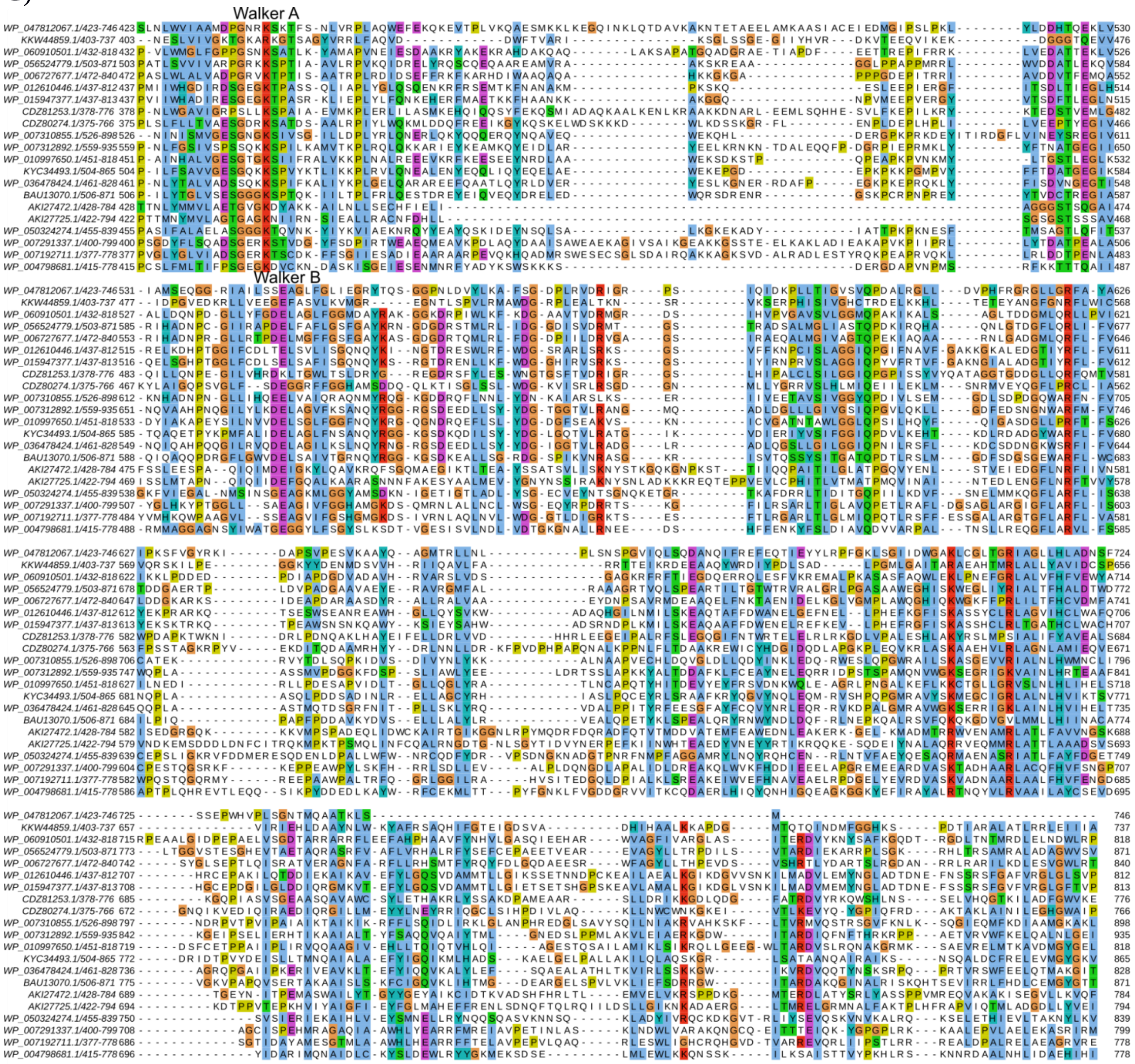

D)

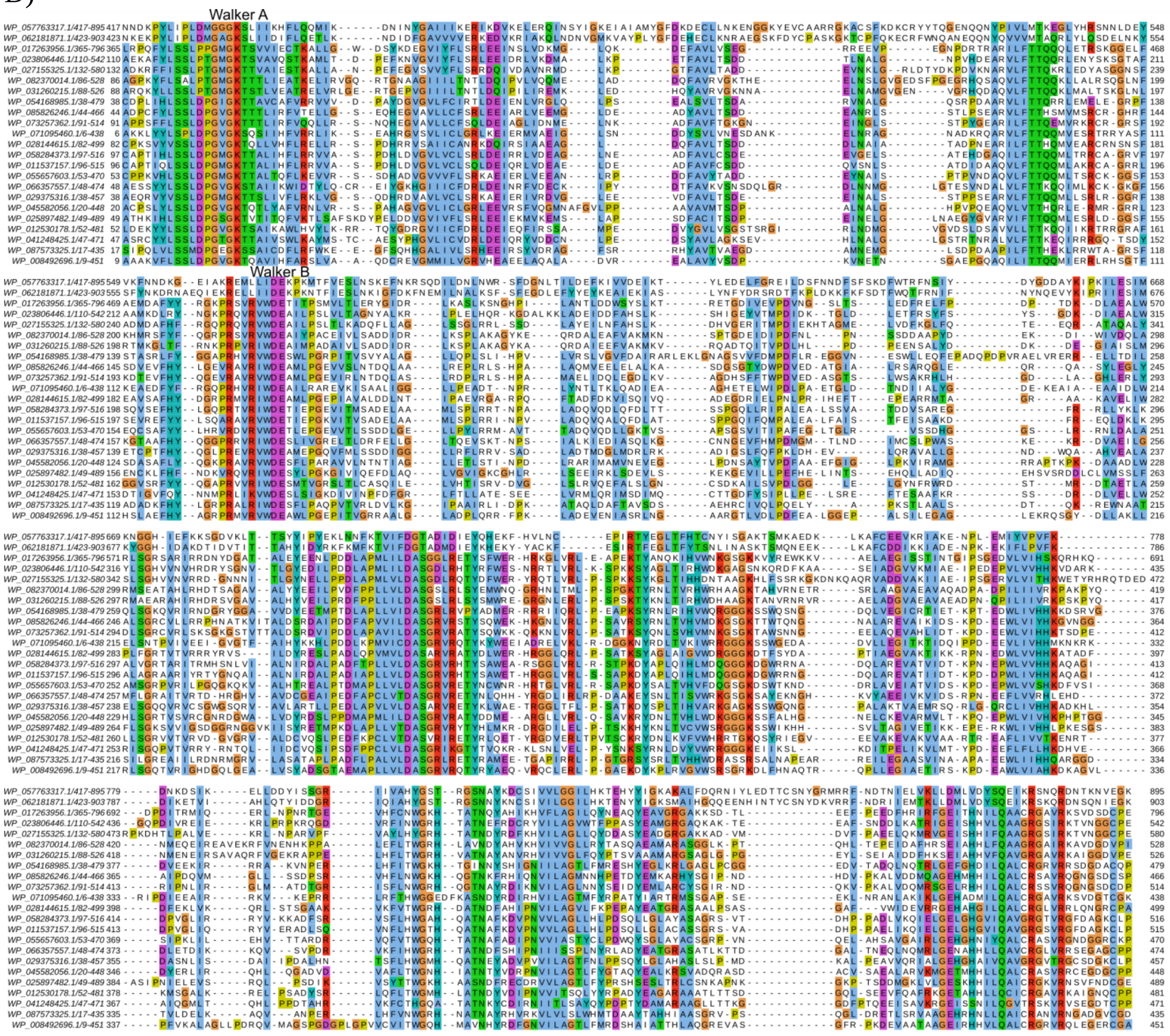

Figure S5. Sequence alignments of putative new helicases. A. VirE family helicases. B. DUF927 family helicases. C. DUF3987 family helicases. D. pVOG1685/pVOG9714 family helicases. Walker A and B motifs are indicated.

```
>PRK02249 DNA primase large subunit; Validated
Probab=94.98  E-value=0.21  Score=46.31  Aligned_cols=90  Identities=18%  Similarity=0.355  Sum_probs=62.1

Q ss_pred               HHHHHh---cCCCCCHHHHHHHHHhhcccc-ChhHHHHHHHhCCCCCCCHHHHHHHHHH-hhc-----CCCCchhhhhhhc
Q WP_063441057      51 IKSCVD---HQNDVGYFEWISMLQTVLHCE-NGENYAHKWSSEHDEYDESYTNLKLQY-LSE-----YAPVTCERVEKEL  120 (145)
Q Consensus        51 ~~~c~~----~a~~lsEP~W~amis~la~~~~-dG~~~~He~S~~yp~Ys~~ETd~Ki~~-l~-------~gP~TC~~I~~~~~  120 (145)
                        |+++.+   ....|+...=+++.+-|..+. +=++++.-|+ .-|.|++.+|.--|+| ...      ..|.+|++|+. +
T Consensus      226 m~~l~~~l~~g~~L~h~~rf~l~~FL~~iG~~~dei~~~~~-~~~~f~e~~~~Y~i~h~~G~~G~~~Y~ppsC~~m~s-~  303 (343)
T PRK02249       226 MKALLSALQAGENLPHTARFAITSFLLNIGMSVDEIVELFR-NAPDFDEEKTRYQVEHIAGETGGTEYTPPSCETMRT-Y  303 (343)
T ss_pred               HHHHHHHHhcCCCCChHHHHHHHHHHHhcCCCHHHHHHHh-cCccccchhhHHhhhhhccCCcCCccccCCCChHHHHh-C
Confidence             555443   4688888888888888777774 3345555554 4588999999888888 432      33999999995 5


Q ss_pred               CCCCCCC-CCCCCCcCCcccccccc
Q WP_063441057     121 GCEHCIN-CPFKNEVNTPLTLGFWK  144 (145)
Q Consensus       121 G~~~C~~~-Cp~~~~~ikSPi~Lg~~k  144 (145)
                        |.   |.+ |..=..|++|++++.++
T Consensus      304 g~--C~~~d~~C~~i~hPL~yy~~~  326 (343)
T PRK02249       304 GL--CVGKDDLCEKVKHPLSYYRRK  326 (343)
T ss_pred               CC—CCCcccccccCCCChHHHHHHH
```

Figure S6. Alignment of profiles of PriCT-2 (PrimQH_Bbadius, accession: WP_063441057) and primase large subunit (CDD: PRK02249). Conserved cysteine residues are shown in yellow background.
